# Application of Coltosol F with a cotton-spacer technique for endodontic temporization: a clinical retrospective study

**DOI:** 10.1186/s12903-026-07988-2

**Published:** 2026-03-13

**Authors:** Andrea Spinelli, Carlo Prati, Maria Giovanna Gandolfi, Fausto Zamparini

**Affiliations:** 1 Endodontic Clinical Section, Dental School, Department of Biomedical and Neuromotor Sciences, University of Bologna, Bologna, Italy; 2 Laboratory of Green Biomaterials and Oral Pathology, Dental School, Department of Biomedical and Neuromotor Sciences, University of Bologna, Bologna, Italy

**Keywords:** Coltosol F, Endodontic temporization, Temporary restorative materials, Cotton pellet spacer, Tooth fractures

## Abstract

**Background:**

Temporary restorative materials are important for maintaining coronal seal during multi-visit endodontic procedures. Coltosol F, a moisture-activated, self-setting material, is valued for its simplicity and sealing ability, though concerns exist regarding its potential to induce tooth fractures.

**Objective:**

To evaluate the clinical performance of Coltosol F as temporary restoration material, focusing on tooth fracture incidence, spontaneous dislodgement and the role of a cotton pellet as a spacer material. DuoTemp placed with a cotton pellet and Coltosol F placed without a cotton pellet were used as control groups.

**Materials and methods:**

A retrospective analysis of 153 consecutive patients requiring primary endodontic treatments (n = 185) performed in an endodontic clinical section from 2019 to 2024 was conducted. All treatments were performed by postgraduate master operators through a predefined rehabilitation protocol. A total of 111 patients, corresponding to 138 Coltosol F treated teeth, were analyzed: 107 teeth with a cotton pellet used as a spacer material (Group A) and 31 without (Group B). Additional groups included 15 patients with 18 teeth provisionally restored with Coltosol F and a cotton pellet under a provisional crown (Group C), and 27 patients with 29 teeth provisionally restored with DuoTEMP (Group D). Outcomes evaluated after 7–14 days included coronal fractures, root fractures and material dislodgment.

**Results:**

At 7–14 days, adverse events were rare. In the Group A with cotton pellet group, we observed 3 coronal fractures (2.8%), 1 root fracture (0.9%), and 5 spontaneous dislodgements (4.6%). In the Group B, there were 1 coronal fracture (3.2%), 1 root fracture (3.2%), and 1 dislodgement (3.2%). Group C had no adverse events. Group D showed 1 coronal fracture (3.4%) and no dislodgement. Between-group differences were not statistically significant.

**Conclusion:**

Coltosol F showed a low short-term incidence of fracture and dislodgement during 7–14-day inter-appointment temporization. No statistically significant differences were detected between groups. No adverse events were observed when the temporary restoration was shielded by a provisional crown.

## Introduction

Multi visit endodontic treatment requires a temporary restoration that seals the access cavity, resists masticatory forces and prevents salivary and microbial contamination until the final restoration is placed. Microleakage through a poorly sealed access cavity can lead to reinfection and failure of the therapy. Therefore, temporary materials should provide a minimum of 3/4-mm thickness, low porosity, dimensional stability, and mechanical durability. Ideally, they should also offer effective marginal sealing to prevent bacterial infiltration, ease of manipulation, a quick setting time, biocompatibility, antibacterial properties, and an easy removal [1]. The concept of endodontic temporization has been comprehensively reviewed by Naoum and Chandler, who highlighted that an adequate coronal seal between appointments is essential to prevent bacterial recontamination and to support the long-term success of root canal treatment [2]. 

A calcium sulphate, zinc oxide–based eugenol-free temporary restorative material (Coltosol F, Coltène/Whaledent AG, Altstätten, Switzerland) is widely used and has been investigated in in vitro studies. It may set by a moisture-activated reaction and exhibits approximately 17–20% hygroscopic expansion [3, 4]. This property enhances its sealing ability by allowing the material to adapt to cavity walls, but it also raises concerns regarding internal stress, especially in structurally weakened teeth [5]. In vitro studies have shown that this expansion may cause cracks and even vertical fractures, particularly when it fills the entire pulp chamber and the endodontic cavity is free from any “spacer” materials [6] .

The role of spacer materials, such as cotton pellets or PTFE, has been investigated in some in vitro and in vivo evaluations [7]. Spacer materials applied into the deeper part of pulp chamber may have a critical function to absorb liquid from root canals and may indicate the presence of infected fluids, but may also modulate stress distribution, facilitate re-opening of the cavity and new detection of root canal orifices. Robust evidence establishing the superiority of any spacer material is lacking.

This study investigates the clinical performance of different provisional restoration techniques when used as an inter-appointment temporary material in endodontic therapy. Specifically, coronal fractures, root fractures and material dislodgment were evaluated.

## Materials and methods

### Study design and clinical setting

This was a retrospective, observational clinical study conducted in a University Endodontic department. Patients were treated by a pool of post graduate students from 2019 to 2024. The study focused on the use of Coltosol F as an inter-appointment temporary endodontic restorative material.

As a retrospective observational study based on consecutive clinical cases, no a priori sample size calculation or power analysis was performed. The sample size was determined by the number of eligible patients treated during the study period. Informed consent was obtained from all patients. All patients were treated according to the principles established by the Declaration of Helsinki as modified in 2013 (Helsinki). The study was approved by the Independent Ethics Committee of Area Vasta Emilia Centro (CE-AVEC), Azienda USL di Bologna, Bologna, Italy (CE-AVEC reference No. 461/2020/OSS/AUSLBO).

The following criteria were used for patient selection and enrolment.

Inclusion Criteria

- good systemic health, defined as ASA I or stable ASA II, eligible for routine endodontic treatment
- Returned for follow-up within 7–14 days.
- At least 2 residual dentinal walls
- Necessity of a primary root canal treatment with a diagnosis of irreversible pulpitis or necrosis

Exclusion Criteria

- patients requiring special medical precautions or modified clinical management
- Parafunctional habits (bruxisms)
- Multiple rehabilitations
- Used as abutment for fixed or mobile prostheses
- Lack of antagonists

### Restorative protocols

#### Materials used

According to the manufacturer’s Safety Data Sheet (SDS) Coltosol F is a radiopaque, moisture-setting temporary restorative material indicated for short-term temporization (up to 1–2 weeks). It is based on zinc oxide (30–<35% w/w) and zinc sulfate cement chemistry (10–<15% w/w) and contains zinc oxide, zinc sulfate monohydrate, calcium sulfate hemihydrate, diatomite, EVA resin, sodium fluoride (< 0.01%), and mint flavor (< 1% w/w). The material sets through water uptake (hygroscopic setting/expansion), with surface hardening in approximately 20–30 min and functional resistance to masticatory load after approximately 2–3 h.

DuoTEMP is a temporary restorative paste that can be used in a self-curing mode and whose setting can be accelerated by light-curing. Based on the SDS, its main constituents include zinc oxide (30–<35% w/w), urethane dimethacrylate (UDMA; 15–<20% w/w), zinc sulfate monohydrate (10–<15% w/w), and mint flavor (< 1% w/w).

Cotton pellets used as spacer were sterile medical-grade cotton/cellulose pellets placed dry in the pulp chamber as described in the operative protocols.

#### Clinical procedures

All patients included required a primary root canal treatment for pulpitis or for pulp necrosis. The treatment was performed following a predefined endodontic protocol previously established [8, 9]. 

Four treatment groups were evaluated:

- Group A (*n* = 107 teeth/83 patients): Coltosol F placed over a cotton pellet positioned in the pulp chamber.
- Group B (*n* = 31 teeth/28 patients): Coltosol F placed directly in the access cavity, without a cotton pellet.
- Group C (*n* = 18 teeth/15 patients): Coltosol F placed over a cotton pellet and covered by a previously prepared provisional crown.
- Group D (*n* = 29 teeth/27 patients): DuoTEMP placed over a cotton pellet positioned in the pulp chamber.

All provisional restorations were made at the end of first endodontic section, after final irrigation with NaOCl and paper point application. All treatments were performed under rubber-dam isolation. The restorative aim was to achieve a minimum occlusal thickness of 3/4 mm of temporary material.

Group A. After final irrigation and drying with paper points, sterile cotton pellets were placed passively in the pulp chamber to cover the canal orifices. In molars, two to three pellets were used; in premolars, one to two, sized to fill the pulp chamber while preserving 3/4 mm for the temporary material. The pellets were placed dry. ROEKO^®^ cotton pellets were used; when unavailable, cotton pellets from other manufacturers were employed. Coltosol F was then placed and condensed using a spatula instrument to adapt to the cavity walls. The surface was than moistened to initiate its hygroscopic, moisture-activated set. After the initial set (≈ 3–5 min), minor excess was removed. Occlusion was checked with articulating paper (~ 40 μm) and premature contacts were removed. Patients were advised to avoid hard foods and to reduce occlusal load until the definitive restoration.

Group B. The same protocol was followed. No intermediate material was placed in the chamber.

Group C. After cleaning and drying as described a cotton pellet was positioned in the pulp chamber and Coltosol F was adapted within the endodontic access. Following moisture activation and initial set, excess material was removed, and interferences were excluded. The previously prepared provisional crown was then temporarily cemented according to routine clinical practice, excess cement was removed, and occlusion was verified.

Group D. DuoTEMP was applied into the access cavity and adapted to achieve a ≥ 4 mm occlusal thickness following the manufacturer recommendations. Setting was accelerated by light curing, and occlusion was checked and refined.

### Outcome measures

Patients were reassessed after 7–14 days. Recorded outcomes were:

- coronal fractures,
- root fractures
- material dislodgment or marginal deterioration

Tooth status was checked at each appointment and any complication (material dislodgment, fractures) was recorded. Occurrence of vertical root fractures (events that compromise the treatment and final outcome) were confirmed by clinical and radiological signs, such as localized deep probing, localized pain upon biting or radiographical visualization.

### Statistical analysis

Comparisons of complication rates between groups were performed using contingency table methods. Because of small sample sizes in some groups, Fisher’s exact test was used for pairwise comparisons, while a chi-square test of independence was applied to assess overall group differences.

## Results

Number of treated teeth and group constitutions, as well as the demographic characteristics of the cohort is reported in Tables 1and 2. A total of 185 teeth were included and distributed among the four temporary-restoration groups: Group A (*n* = 107), Group B (*n* = 31), Group C (*n* = 18), and Group D (*n* = 29). The demographic characteristics of the cohort were balanced across groups. Males and females were similarly represented, with only minor variations in proportions. Tooth-related variables also showed similar patterns: maxillary and mandibular teeth were evenly represented, molars constituted the majority in all groups, and premolars and anterior teeth occurred less frequently. Pulpitis was the predominant diagnosis across all groups, ranging from 66.6% to 84.2%.

Table 3 reports the clinical outcomes at 7–14 days. In Group A (*n* = 107), we recorded 3 coronal fractures (2.8%), 1 vertical root fracture (0.9%), and 5 spontaneous dislodgements (4.6%). The vertical root fracture in Group A occurred in a maxillary molar. The three coronal fractures involved one maxillary molar, one mandibular molar, and one maxillary premolar. In Group B (*n* = 31), we observed 1 coronal fracture (3.2%), 1 vertical root fracture (3.2%), and 1 dislodgement (3.2%); both the coronal fracture and the vertical root fracture occurred in a mandibular molar. Group C (*n* = 18) showed no adverse events. Group D presented 1 coronal fracture and no dislodgements.

**Table 1:** Demographic characteristic of the cohort

		Group A *n* (%)	Group B *n* (%)	Group C *n* (%)	Group D *n* (%)
Gender	Males	49 (45.7)	16 (51.1)	11 (61.1)	11 (37.9)
	Females	58 (54.2)	15 (48.3)	7 (38.8)	18 (62.1)
Age	< 30	40 (37.3)	11 (35.4)	4 (22.2)	11 (37.9)
	30–55	28 (26.1)	14 (45.1)	7 (38.8)	8 (27.5)
	> 55	39 (36.4)	13 (41.9)	7 (38.8)	10 (34.4)
Location	Maxilla	62 (57.9)	13 (41.9)	8 (44.4)	15 (51.8)
	Mandible	45 (42.05)	18 (58.1)	10 (55.5)	14 (48.2)
Tooth type	Anterior	18 (16.8)	6 (25.8)	4 (22.2)	3 (10.3)
	Premolars	25 (23.3)	8 (51.1)	8 (44.4)	11(37.9)
	Molars	64 (59.8)	17 (54.8)	6 (33.3)	15 (51.7)
Diagnosis	Pulpitis	90 (84.2)	25 (80.6)	12 (66.6)	23 (79.4)
	Pulp Necrosis	17 (15.8)	6 (19.3)	6 (33.3)	6 (20.6)
	Total	107	31	18	29

**Table 2:** Number of treated teeth and group constitutions. Complications are reported as n (%)

Group	Group A *n* (%)	Group B *n* (%)	Group C *n* (%)	Group D *n* (%)
Cases	107	31	18	29
Complications	9 (8.4%)	3 (9.6%)	0	1 (3.4%)

**Table 3:** Types of complications within groups. Vertical fractures are accounted as fatal complications, leading to tooth extraction

Group	Coronal Fractures *n* (%)	Root Fractures *n* (%)	Spontaneous dislodgments *n* (%)
Group A (*n* = 107)	3 (2.8%)	1 (0.9%)	5 (4.6%)
Group B (*n* = 31)	1 (3.2%)	1 (3.2%)	1 (3.2%)
Group C (*n* = 18)	0	0	0
Group D (*n* = 27)	1 (3.4%)	0	0

The overall chi-square test across all four groups did not show significant differences (χ^2^ = 4.01, df = 3, *p* = 0.261). Pairwise comparisons likewise yielded no statistically significant results. For example, Group A showed a lower complication rate Group B (*p* = 0.50), whereas Group A appeared to have a higher complication rate than Group D (*p* = 0.46). Comparisons involving the group C were constrained by the absence of events, producing infinite or zero odds ratios but nonsignificant p-values (all *p* > 0.14).

**Fig. 1:**
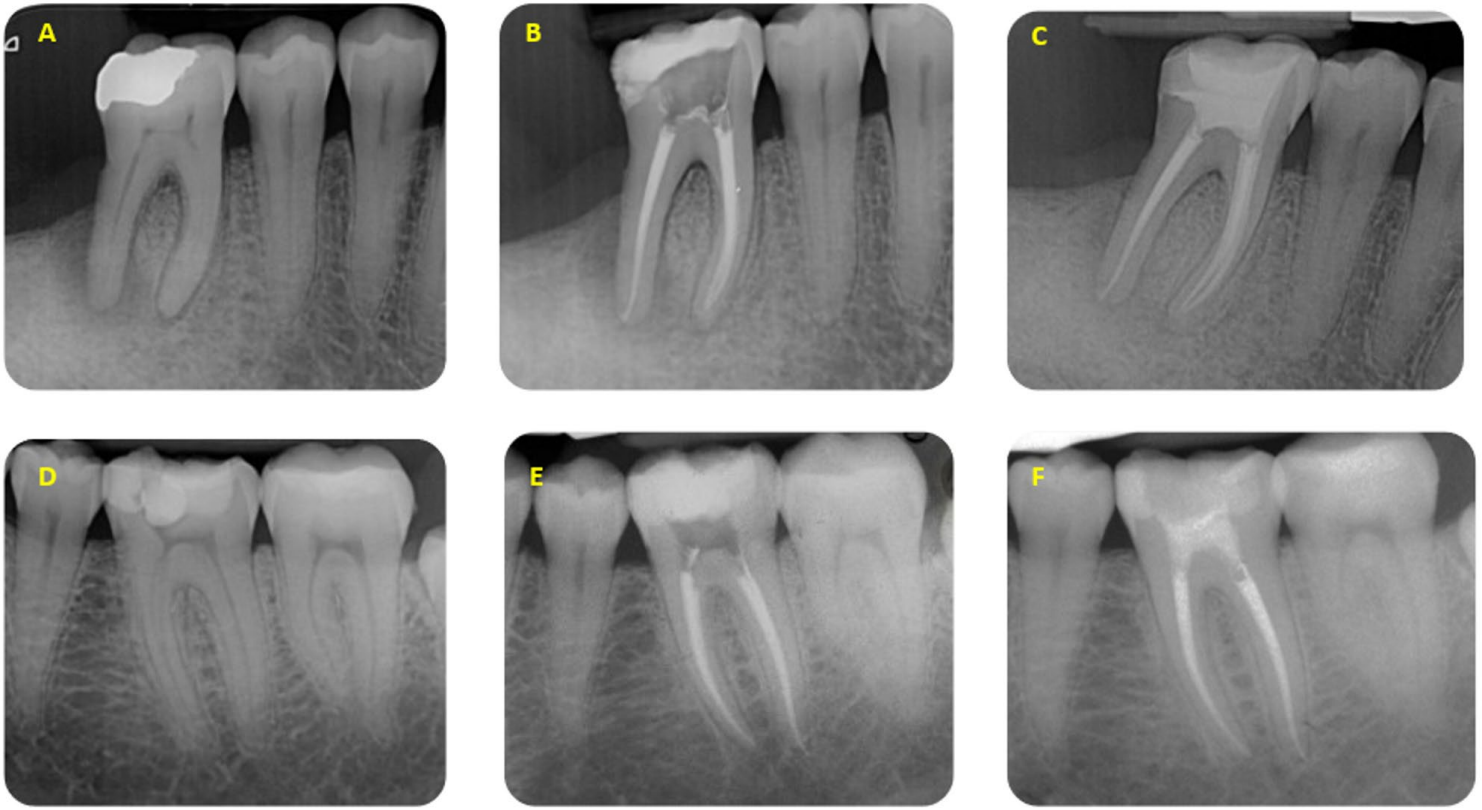
Representative periapical radiographs of posterior teeth with Coltosol F used as a temporary restoration during multi-visit endodontic treatment. **A** Preoperative radiograph of a 4.6 showing a periradicular lesion on distal root. **B** root canals filling with Thermafil. Please note the access cavity sealed with an approximately 3–4 mm occlusal plug of Coltosol F placed over a cotton pellet. Residual dentinal walls are preserved and no cracks are detectable. **C** Final radiograph after definitive adhesive restoration, showing maintenance of coronal integrity and absence of radiographic signs of cusp deflection or root fracture. **D** Pre-operative radiograph of a 3.6 showing a deep carious lesion. **E** Immediate post-operative radiograph after root canal obturation performed with Thermafil; the access cavity was temporarily sealed with a 3–4 mm layer of Coltosol F placed over a cotton pellet used as a spacer material to provide coronal seal before definitive restoration. **F** Radiograph after removal of the temporary material and placement of the final post-endodontic restoration, with intact coronal morphology and no radiographic evidence of fractures or material loss. None of the teeth illustrated experienced spontaneous dislodgement or radiographically or clinically detectable complications

Two representative cases included in the study are reported in Fig. 1.

## Discussion

This clinical study demonstrates a low overall incidence of short-term mechanical complications, including coronal fractures, vertical root fractures, and material dislodgements, across all evaluated temporization protocols. These early mechanical events were the primary focus of the present investigation, as they may directly compromise tooth integrity and potentially affect treatment outcome [8]. Other biologically relevant outcomes, such as microbial leakage, reinfection, and periapical healing, were not evaluated in this study and therefore cannot be inferred from the present findings.

Our findings must be interpreted in light of previous literature, which has reported higher rates of fractures and marginal degradation for Coltosol than what we observed.

Djouiai and Wolf reported in an in vitro study with micro-CT a 40% fracture rate in Coltosol-filled teeth after 15 days [10]. While micro-CT allows for highly sensitive detection of microdefects, it may also introduce artifacts or reveal damage not clinically relevant. Similarly, Tennert at al using a stereomicroscope with a 10x magnification observed a 39% of tooth structure fracture when Coltosol was placed in mesio-occlusal-distal cavities without an intermediate spacer [6]. Sample preparation may alter dentin integrity, limiting the clinical applicability of in vitro findings; although useful for standardization, these evaluations do not fully replicate the clinical environment, where occlusal load, hydration dynamics, and cavity design may influence outcomes. This data is significantly higher than percentage reported in our cohort. Our clinical findings showed a low fracture incidence compared with several in vitro reports. We reported two vertical root fractures, one occurring in upper molar and one in lower molar (fatal events leading to extraction). The predominance of fractures in molars is clinically plausible given the higher occlusal loads and the typically larger access cavities/cuspal involvement in posterior teeth. Importantly, most fractures in our cohort were coronal and did not compromise the endodontic therapy. Episodes of material dislodgement were rare and did not affect treatment outcome; in such cases, before proceeding, we dedicated additional time to re-establish canal disinfection with endodontic irrigants.

Spacer materials also play a key role. Solomonov et al. [7] demonstrated that PTFE spacers allowed greater bacterial penetration (*E. faecalis*) than cotton pellets under Coltosol or IRM. Beyond mechanical buffering, the absorbency and conformability of cotton likely contribute probably to better marginal adaptation. In contrast other in vitro study reports that cotton fibres can absorb fluids and act as a reservoir for bacteria, compromise the peripheral seal and reduce the effective thickness of the temporary material [1]. A micro-computed tomography study comparing spacer materials found that cotton pellets increased the gap between the restoration and dentine walls, whereas polytetrafluoroethylene (PTFE) spacers maintained a tighter seal with fewer gaps [11].

This concern has been discussed in the literature, and comparative studies have suggested differences among spacer materials (e.g., cotton versus PTFE) in terms of microbial penetration and/or interfacial gaps at the restoration–tooth interface [1, 7, 11]. A recent systematic review comparing cotton pellets and polytetrafluoroethylene (PTFE) as endodontic spacers reported consistently lower microbial contamination with PTFE; however, the available evidence was described as scarce and heterogeneous, with widely variable risk of bias across the included studies, limiting the strength of clinical recommendations [12].

In the present cohort, several measures were adopted to mitigate this risk: cotton pellets were placed sterile and dry under rubber dam isolation; a ≥ 3–4 mm occlusal thickness of temporary material was placed and the inter-appointment period was limited to 7–14 days. Moreover, in the few cases in which spontaneous dislodgement occurred, additional time was dedicated to re-isolation and decontamination of the pulp chamber (and, when indicated, supplementary canal re-irrigation/disinfection) before proceeding with the subsequent appointment, in order to minimize the impact of possible coronal leakage. As part of the post-endodontic protocol, the coronal gutta-percha was cut back to the level of the bone margin or 1–2 mm apical to root canal orifices, with the dual aim of improving the quality of the coronal seal in the restorative phase and removing potentially contaminated coronal filling material.

While fractures and dislodgements represent clinically evident adverse events, the most critical endpoint of inter-appointment temporization in endodontics is the ability to maintain an effective coronal seal and minimize microleakage-driven (re)contamination of the root canal system, which may contribute to persistent intraradicular infection and periapical inflammation. Therefore, the low fracture incidence observed in our cohort should be interpreted specifically as a short-term mechanical finding, since microbial leakage can occur subclinically without immediate mechanical failure. In this study, outcomes were intentionally limited to short-term mechanical events over a 7–14-day interval, and periapical inflammatory outcomes or direct leakage measurements were not assessed. Microbial leakage/biofilm regrowth was not directly assessed. These approaches have been adopted in pre-clinical research to better characterize the coronal seal of temporary restorations and the spacer–restoration interface showing lower contamination when PTFE is used instead of cotton in some experimental settings [13]. Likewise, micro-CT has been applied to assess the sealing ability of temporary restorative materials and to quantify interfacial gaps/porosity, including studies reporting that spacer choice (cotton vs. PTFE) can influence gap formation [11]. Additional laboratory work has evaluated bacterial/glucose microleakage across different temporary restoration strategies [14].

Interestingly, clinical practice patterns suggest that cotton pellets remain the most commonly used spacer material: in a survey of Diplomates of the American Board of Endodontists, 83% of respondents reported routinely placing a cotton pellet beneath temporary restorations [15]. Our findings therefore provide clinically relevant data on a temporization strategy that is widely adopted in everyday endodontic practice.

Studies by Milani et al., Naseri et al., and Shahi et al. [16–18] all suggest that Coltosol is susceptible to marginal degradation within 7–14 days. In vitro model often relies isolated mechanical setting that do not fully capture the complex oral environment. The absence of clinical symptoms in our patients despite potential subclinical degradation supports the notion that microleakage observed in vitro may not always associate to clinical failure.

Structural factors are also relevant; Balkaya et al. [4] demonstrated that temporary filling material did not affect the fracture resistance suggesting that cavity design was more effective than temporary material choice in determining fracture risk.

While our results show better clinical outcomes than many in vitro and in vivo studies, this may be attributed to the integration of clinical strategies such as cotton pellet used as a spacer material, occlusal adjustment, and short-term temporization. Nevertheless, further randomized clinical trials and real-time microbial leakage analyses are needed to validate these findings and define evidence-based temporization protocols.

Although our protocol did not aim to induce biomineralization, experimental ZnO-modified hydroxyapatite cements have shown, within 21 days, precipitation of both crystalline-stoichiometric and amorphous apatite on dentin, accompanied by increases in phosphate Raman signals, tubule occlusion, and enlargement of collagen fibril width. Mechanistically, Zn substitution is associated with enhanced bioactivity and may also inhibit matrix metalloproteinases while promoting collagen cross-linking, potentially stabilizing the dentin–material interface during temporization [19].

As a final consideration based on our clinical studies published over the years [8, 9, 20–22], the same technique has been routinely adopted in our department. Across these cohorts, only few fractures and complications were reported. These studies support the routine use of a cotton pellet with Coltosol F while we await larger randomized trials to further validate and refine evidence-based endodontic inter-appointment strategies.

The present study has several limitations: the study design was observational and non-randomized, introducing potential selection bias; microbial leakage was not quantitatively assessed, and clinical evaluation of marginal adaptation was visual and not instrumentally verified, therefore, minor marginal discrepancies or early interfacial degradation may have been underestimated;. Future prospective studies should incorporate objective and standardized assessments of marginal adaptation (e.g., calibrated scoring systems under magnification, photographic documentation, and/or validated leakage or imaging methods) to better correlate marginal integrity with dislodgement risk and microbiological outcomes. A further limitation is that the amount of remaining coronal tooth structure (cusp thickness/volume) was not quantitatively measured or recorded However, we attempted to standardize the baseline structural condition through the inclusion criteria. Specifically, only teeth presenting at least two residual dentinal walls were included in the study. This criterion was adopted because recent studies indicates that the presence of ≥ 2 residual coronal walls is a relevant prognostic factor for endodontically treated teeth, significantly influencing long-term survival and biomechanical behavior [23]. Previous investigations also showed that teeth with severe structural loss (e.g., < 30% of original coronal tooth structure) exhibit a significantly reduced prognosis [24].

Future culture/qPCR studies could explore pre-wetting the cotton pellet with disinfectant solutions in selected cases such as necrotic pulps or chronic apical periodontitis to provide an immediate antimicrobial reservoir at the chamber floor. Promising agents (e.g., chlorhexidine or iodine-based solutions, green tea, ozone, antibiotic etc.) might reduce intra coronal bacterial load [25–27].

Future studies should incorporate randomized controlled trials and quantitative analyses of marginal integrity over time. In vivo microbial leakage models and advanced imaging methods could further elucidate the sealing capabilities and degradation patterns of temporary materials. Until such evidence is available, the dry-pellet approach adopted in this study remains a prudent baseline protocol.

## Conclusions

Within the limitations of this retrospective observational study, inter-appointment temporization over 7–14 days was associated with a low incidence of mechanical adverse events (coronal fracture, vertical root fracture, and spontaneous dislodgement) across all evaluated protocols. Cotton pellet showed lower but non statistically significant differences when compared to other groups. The group shielded by a provisional crown showed no adverse events in this cohort at the same observational time.

## Data Availability

Data available upon reasonable request.

## Abbreviations

ASA: American Society of Anesthesiologists (physical status classification)
EVA: Ethylene–vinyl acetate
IRM: Intermediate restorative material
micro-CT: Microcomputed tomography
NaOCl: Sodium hypochlorite
PTFE: Polytetrafluoroethylene
qPCR: Quantitative polymerase chain reaction
SDS: Safety Data Sheet
UDMA: Urethane dimethacrylate
ZnO: Zinc oxide
